# Radiotherapy modulates expression of EGFR, ERCC1 and p53 in cervical cancer

**DOI:** 10.1590/1414-431X20176822

**Published:** 2017-11-13

**Authors:** V.H. de Almeida, A.C. de Melo, D.D. Meira, A.C. Pires, A. Nogueira-Rodrigues, H.K. Pimenta-Inada, F.G. Alves, G. Moralez, L.S. Thiago, C.G. Ferreira, C. Sternberg

**Affiliations:** 1 Divisão de Pesquisa Clínica e Desenvolvimento Tecnológico, Instituto Nacional de Câncer, Rio de Janeiro, RJ, Brasil; 2 Instituto de Bioquímica Médica Leopoldo De Meis, Universidade Federal do Rio de Janeiro, Rio de Janeiro, RJ, Brasil; 3 Departamento de Ciências Biológicas, Universidade Federal do Espírito Santo, Vitória, ES, Brasil; 4 Fonte Medicina Diagnóstica, Niterói, RJ, Brasil

**Keywords:** Cervical cancer, Radiotherapy, Epidermal growth factor receptor (EGFR), p53, Excision repair cross-complementation group 1 (ERCC1)

## Abstract

Cervical cancer is a public health problem and the molecular mechanisms underlying radioresistance are still poorly understood. Here, we evaluated the modulation of key molecules involved in cell proliferation, cell cycle and DNA repair in cervical cancer cell lines (CASKI and C33A) and in malignant tissues biopsied from 10 patients before and after radiotherapy. The expression patterns of epidermal growth factor receptor (EGFR), excision repair cross-complementation group 1 (ERCC1) and p53 were evaluated in cancer cell lines by quantitative PCR and western blotting, and in human malignant tissues by immunohistochemistry. The mutation status of *TP53* gene was evaluated by direct sequencing. Among cell lines, absent or weak modulations of EGFR, ERCC1 and p53 were observed after exposure to 1.8 Gy. Conversely, increased expressions of p53 (5/10 patients; P=0.0239), ERCC1 (5/10 patients; P=0.0294) and EGFR (4/10 patients; P=0.1773) were observed in malignant tissues after radiotherapy with the same radiation dose. *TP53* mutations were found only in one patient. Here we show that a single dose of radiotherapy induced EGFR, ERCC1 and p53 expression in malignant tissues from cervical cancer patients but not in cancer cell lines, highlighting the gap between *in vitro* and *in vivo* experimental models. Studies on larger patient cohorts are needed to allow an interpretation that an upregulation of p53, EGFR and ERCC1 may be part of a radioresistance mechanism.

## Introduction

Cervical cancer is a public health problem representing the fourth most common cancer in women worldwide, with more than 500,000 new cases per year (1). The combined treatment, involving cisplatin-based chemotherapy and radiotherapy, has been established as the standard therapeutic approach for patients with locally advanced disease. Radiotherapy alone has also been used for patients with early disease (2). However, patients with stage III and IVA tumors have 5-year survival rates lower than 50% (3), and novel strategies to improve the prognosis of these patients are needed. An increased understanding of the molecular mechanisms leading to radioresistance may offer novel radiosensitizing strategies.

Virtually all cervical cancers (99% or more) are caused by high-risk human papillomavirus (HPV) (4). E6 and E7 HPV oncoproteins have been known to play a major role in malignant transformation of epithelial cells, mainly by inhibiting p53 and Rb tumor suppressor proteins. On the other hand, the role of the viral E5 protein remains poorly understood. Some studies indicate that E5 may increase epidermal growth factor receptor (EGFR) recycling to the cell surface and enhance growth factor signal transduction (5). Indeed, previous reports have shown EGFR to be frequently expressed in cervical cancer (around 80%) and correlated with disease progression (6,7). EGFR belongs to the HER tyrosine kinase receptor family and EGFR activation leads to dimerization, autophosphorylation and activation of downstream signaling pathways which regulate cell proliferation, survival and transformation (8).

The most critical DNA lesion induced by radiation is DNA double-strand breaks. In mammalian cells, these lesions are predominantly repaired by non-homologous end joining (NHEJ), which protein machinery is mainly composed by DNA-dependent protein kinase (DNA-PK), Ku-70 and Ku-80 (9). Beyond a critical role in DNA repair, activated DNA-PK also phosphorylates proteins involved in cell cycle checkpoints and cell death pathway, such as p53 (10). However, in most cervical cancers, inactivation of p53 is mainly caused by HPV infection (11). Recently, excision repair cross-complementation group 1 (ERCC1) protein also was described to facilitate DNA repair of double-strand breaks induced by radiotherapy (12).

Genotoxic stress can induce EGFR autophosphorylation, activating signaling pathways that promote proliferation and survival, but EGFR can also modulate DNA damage response by interacting with components of the DNA repair machinery (13). Ionizing radiation induces EGFR internalization to nucleus, association of EGFR with DNA-PK, enhancing kinase activity of the DNA-PK complex and increasing the repair of DNA strand breaks (14). In addition, EGFR can also modulate positively ERCC1 expression in prostate cancer cell lines (15) and physically interact with ERCC1 (16), suggesting a new role for EGFR in DNA repair independent of DNA-PK. Indeed, combination of radiotherapy and EGFR inhibitors can improve local tumor control compared to irradiation alone and this strategy has been introduced into clinical radiotherapy practice (17). Preclinical data from our group showed that cetuximab, a monoclonal antibody anti-EGFR, can sensitize gynecological cancer cell lines to chemoradiation (18).

In the current study, we have evaluated the radiation-induced modulation of EGFR, ERCC1 and p53 in cervical cancer cell lines and in malignant tissues from 10 patients diagnosed with cervical cancer.

## Material and Methods

### Cell lines and irradiation

CASKI and C33A cells were cultured in RPMI 1640 medium (Thermo Fisher Scientific, USA) supplemented with 10% fetal bovine serum and incubated in a humidified atmosphere at 37°C in 5% CO_2_. Cell line authentication via STR profiling was performed to confirm their identity. In addition, cells were tested periodically for mycoplasma contamination by Mycosensor PCR assay (Agilent, USA). For radiotherapy, cells were exposed to γ-rays from a ^60^Co source (Theratron 780C; Theratronics International Limited, Canada) at a dose rate of 2.0 Gy/min.

### Clonogenic assay

In order to establish the radiosensitivity profile of each cell line, cells were seeded onto 12-well plates at a concentration of 150 cells per well. After 24 h, cells were irradiated (2, 4, and 6 Gy) and incubated for 72 h. Cells were then washed with PBS and allowed to proliferate in fresh medium for 10 more days. Colonies were stained with 0.1% crystal violet.

### Western blot analysis

Cells were incubated in serum-free medium for 4 h followed by irradiation (1.8 Gy). After 24h, cells were lysed and 50 µg of proteins from each sample were run on a 6–12% SDS-PAGE gel and transferred to a PVDF Hybond-P (GE Healthcare, Brazil) membrane. Membranes were incubated with antibodies against EGFR (1:500; #2232, Cell Signaling Technology, USA), ERCC1 (1:100; 8F1 clone, Thermo Fisher Scientific, USA), p53 (1:500; G59–12 clone, BD Pharmingen, USA) and HSC70 (heat shock cognate 70 protein; 1:5000; Santa Cruz Biotechnology, USA). After incubation with secondary antibodies, immunoblots were detected using the ECL reagent (GE Healthcare, Brazil). Predicted molecular weights for EGFR, ERCC1, p53, and HSC70 are 170, 39, 53, and 70 kDa, respectively.

### Gene expression analysis

Cell lines were incubated in serum-free medium for 4 h followed by irradiation (1.8 Gy). After 24 h of treatment, total RNA was extracted using TRIzol Reagent (Thermo Fisher Scientific). From each sample, 1.0 μg of total RNA was reverse transcribed to cDNA. Then, quantitative PCR was performed on the aliquots of the cDNA using Taqman Fast Real-Time PCR Master Mix (Thermo Fisher Scientific). The gene expression profile was evaluated using the ABI PRISM 7500 Fast Real-Time PCR System (Thermo Fisher Scientific). The Taqman gene expression assay references are Hs01076078_m1 (*EGFR*), Hs00157415_m1 (*ERCC1*), Hs00153349_m1 (*TP53*) and 4326317E (*GAPDH*). The 2^−ΔΔCT^ method was utilized to analyze the fold increase.

### Patient eligibility

Ten patients diagnosed with stage IIB-IVA cervical squamous cell carcinoma, age 18–70 years, and clinical indication for radiotherapy were enrolled. All patients provided written informed consent before participation. The study was approved by the Institutional Review Board (CEP-INCA) under registry 143/09 and followed the Helsinki Declaration.

### Clinical study design

A prospective trial included locally advanced cervical squamous cell carcinoma patients. Briefly, all 10 patients were treated with teletherapy - 45Gy divided into 25 fractions, which means that each fraction is equivalent to 1.8 Gy. This dose was the same used in cell culture experiments previously described. A tumor biopsy was performed before treatment and a second biopsy was taken 24 h after the first radiotherapy fraction. Cisplatin, when indicated, was prescribed afterwards. The expression pattern of EGFR, ERCC1, and p53 was compared between malignant tissues before treatment and after the first teletherapy with 1.8 Gy dose.

### Tissue microarray (TMA) and immunohistochemistry

For immunohistochemistry, TMA was constructed, as described by Pires et al. (19). All samples were fixed in 10% formalin and embedded in paraffin. Serial TMA sections were cut, mounted on glass slides and dried at 56°C before dewaxing in xylene and rehydration in alcohol. All sections were subjected to heat-induced epitope retrieval in citrate buffer followed by inhibition of endogenous peroxidase (peroxidase block, RE7101, Novocastra, UK). Incubation of primary antibodies against EGFR (D38B1, 1:25, Cell Signaling Technology), ERCC1 (1:100; 8F1 clone, Thermo Fisher Scientific), or p53 (1:100; G59–12 clone, BD Pharmingen) was performed for 1 h. Slides were incubated with NovoLink™ polymer (RE7112, Novocastra), further developed with diaminobenzidine chromogen (RE7105, Novocastra), and finally stained with Mayer hematoxylin, dehydrated and mounted with Canadian balsam. EGFR, ERCC1 and p53 expressions were evaluated semi-quantitatively as positive cells after counting 300–500 tumor cells, being scored as negative (no staining), 1+ (<25% of positive cells), 2+ (25–75% of positive cells) or 3+ (>75% cells staining positively). Negative controls were obtained by omitting the primary antibody.

### TP53 mutation status

Only samples with at least 70% of malignant cells were sent to DNA extraction. Genomic DNA was extracted from paraffin*-*embedded tissues with NucleoSpin FFPE DNA kit (Macherey-Nagel, Germany) following the manufacturer's instructions. TP53 exons 5–9 were amplified by PCR using primers and cycling parameters according to International Agency for Research on Cancer (IARC) protocol (20). The PCR products were then purified with Illustra GFX PCR DNA and gel Band Purification kit (GE Healthcare, Brazil) and directly sequenced in both directions in the ABI 3130XL DNA sequence analyzer (Thermo Fisher Scientific) using the same primers and Big-Dye 3.1 Terminator cycle sequencing chemistry (Thermo Fisher Scientific). The electropherograms were visualized manually on the program Sequencher 4.7 (Gene Codes Corporation, USA).

### Statistical analysis

Quantitative experiments comparing two conditions in cell culture were analyzed by unpaired Student's *t*-test. Immunohistochemistry comparing score intensity between EGFR, p53 and ERCC1 staining before and after radiotherapy was analyzed by paired Student's *t*-test. All statistical analyses were performed using GraphPad Prism Software (USA) and the P values were considered significant when <0.05.

## Results

**Figure 1.**
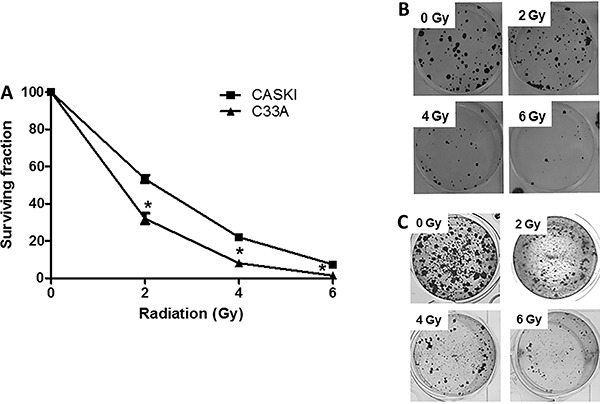
. Radiosensitivity profile of cervical cancer cell lines. *A*, Cells were irradiated (2, 4, and 6 Gy) and incubated for 72 h. Then, cells were washed and allowed to proliferate in fresh medium for 10 more days. The number of colony-forming units in treated cultures is reported as the surviving fraction. *P<0.05 (two-tailed unpaired *t*-test). Representative images of CASKI cells (*B*) and C33A (*C*) cells under different doses of radiation.

We evaluated the radiosensitivity of two cervical cancer cell lines (C33A and CASKI) by clonogenic assay after increasing doses of radiation (2, 4, and 6 Gy). As shown in Figure 1A, C33A was highly sensitive to radiotherapy while CASKI exhibited a radioresistance profile. Representative pictures of colonies of CASKI (Figure 1B) and C33A cells (Figure 1C) exposed to increasing doses of radiotherapy show the dose-response effect.

**Figure 2.**
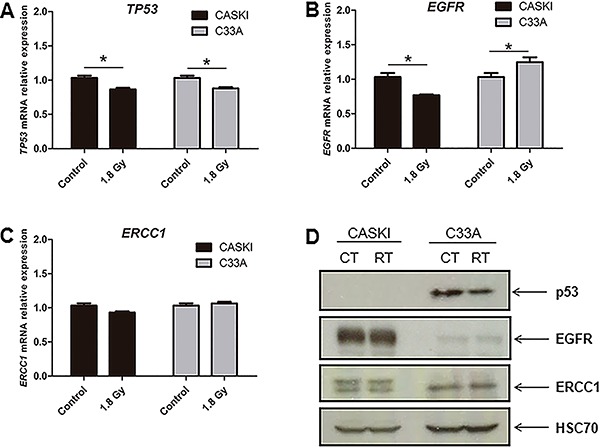
Gene expression modulation induced by radiation in cervical cancer cell lines. Gene expression assays for *TP53* (*A*), *EGFR* (*B*), and *ERCC1* (*C*) were evaluated by quantitative polymerase chain reaction (qPCR). *GAPDH* was used as a housekeeping gene. Cell lines were incubated in serum-free medium for 4 h followed by irradiation (1.8 Gy). After 24 h of treatment, total RNA was extracted, converted into cDNA and the specific sequences amplified by real-time PCR. The relative expression level of mRNA was calculated using the comparative C_T_ method (2^−ΔΔCT^). *P<0.05 (two-tailed unpaired *t*-test). Data are reported as means±SD. *D*, Cells were incubated in serum-free medium for 4 h followed by radiotherapy (1.8 Gy). After 24 h of treatment, cells were lysed and the levels of p53, EGFR and ERCC1 proteins were determined by western blotting. HSC70 was used as a loading control. CT: control, RT: radiotherapy.

Since the dose of each radiation therapy session was 1.8 Gy for each individual patient enrolled in the clinical study, we treated each cell line with the same radiation dose in order to study the expression pattern and compare with the clinical study findings. Overall, absent or weak modulation of p53 (Figure 2A), EGFR (Figure 2B) and ERCC1 (Figure 2C) were observed in cancer cell lines herein investigated at mRNA level. Since the highest radiation-induced variations observed at mRNA levels do not reach 30% and considering that no difference was observed at protein levels (Figure 2D), these slight mRNA variations may have no functional impact. Therefore, independently of the resistance/sensitivity status to radiation therapy, none of the studied cell lines showed important variations at EGFR, ERCC1, and p53 expression profile induced by radiation. It is important to note that the HPV-16 positive CASKI cell line (21) does not present p53 expression (Figure 2D). Finally, although C33A cell line does not exhibit HPV infection (21), it presents *TP53* gene mutation (22).

**Figure 3.**
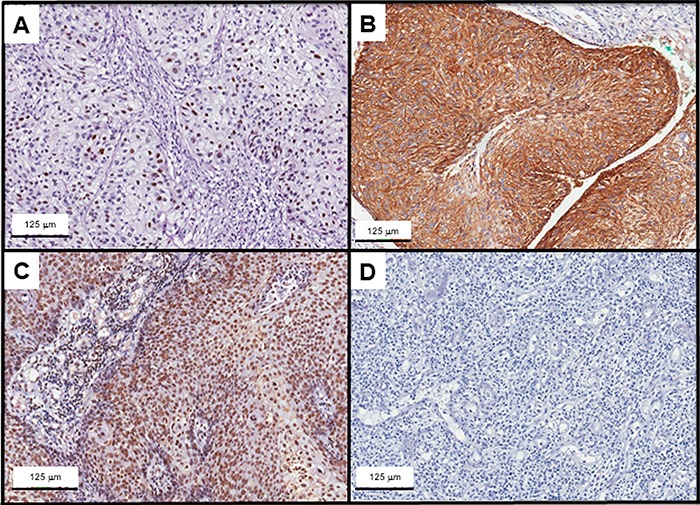
Representative microphotographs of immunohistochemical staining of cervical carcinomas for p53 (*A*), EGFR (*B*), ERCC1 (*C*), and a negative control (*D*). As expected, p53 and ERCC1 staining was confined to the cell nucleus. Original magnification: 100× (scale bar: 125 µm).

The exploratory clinical study herein conducted was intended to confirm and validate the *in vitro* findings. Ten patients, with a median age of 47.5 years (range: 33–66), diagnosed with cervical squamous cell carcinoma were prospectively evaluated in this study. Immunohistochemical analysis of tumor samples before radiation showed positive EGFR staining in 9/10 cases (90%), positive ERCC1 staining in 5/10 cases (50%) and positive p53 staining in 5/10 cases (50%; Table 1). Representative microphotographs of immunohistochemistry for p53 (A), EGFR (B), ERCC1 (C), and an unstained sample (D) are depicted in Figure 3.

**Table 1.** Scoring of p53, EGFR and ERCC1 immunohistochemistry in malignant tissues from cervical cancer patients.

Patients	Before radiotherapy			After radiotherapy		
	p53	EGFR	ERCC1	p53	EGFR	ERCC1
1	-	2+	-	1+	3+	2+
2	-	-	-	2+	3+	1+
3	1+	3+	-	1+	2+	-
4	1+	2+	1+	1+	3+	2+
5	-	2+	3+	1+	3+	3+
6	-	2+	-	-	2+	-
7	-	3+	1+	1+	3+	3+
8	2+	3+	3+	2+	3+	3+
9	1+	3+	-	2+	3+	3+
10	1+	3+	3+	1+	3+	3+

The dose of radiotherapy was 1.8 Gy. Negative score indicates absence of expression in cancer cells; 1+: <25% of positive tumor cells; 2+: 25–75% of positive tumor cells, and 3+: >75% of positive tumor cells.

**Figure 4.**
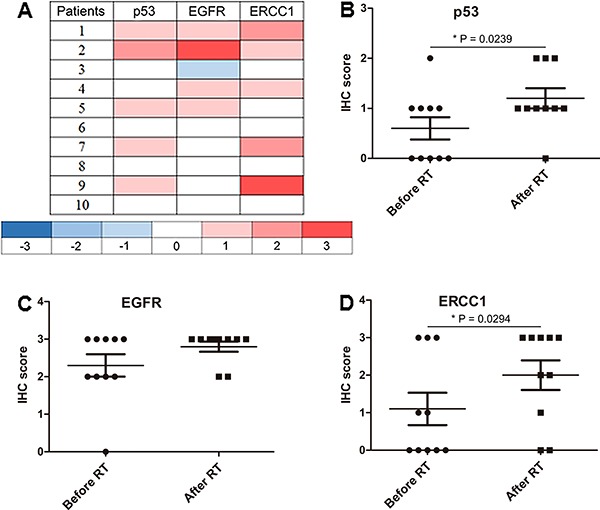
Modulation of p53, EGFR and ERCC1 in malignant tissues from patients submitted to radiotherapy. *A*, Heat map summarizing the modulation of expression of biomarkers for each individual patient before and after one single dose of radiotherapy (RT) (1.8 Gy). Red indicates that radiotherapy increased expression, blue indicates that radiotherapy decreased expression and blank indicates absence of modulation. Comparison of average score of immunohistochemical staining of tumors before and after radiotherapy also was performed for p53 (*B*), EGFR (*C*), and ERCC1 (*D*). *P<0.05 (two-tailed paired *t*-test). IHC: immunohistochemistry.

Our immunohistochemical analysis on tissue microarrays revealed that malignant tissues from 7 of 10 patients exhibited a radiation-induced phenotypic change (Figure 4A). After treatment with 1.8 Gy, an increased expression of p53 as well as of ERCC1 was found in half of the patients while EGFR was positively modulated in 4 of 10 patients (Table 1, Figure 4A). The simultaneous induction of p53 and EGFR protein expression occurred in 3 of 10 patients, while EGFR and ERCC1 was found in 3 of 10 patients; p53 and ERCC1 induction was detected in 4 of 10 patients, while all 3 proteins (EGFR, p53 and ERCC1) were jointly stimulated in 2 out of 10 individuals (Figure 4A). The medium score of p53 staining increased from 0.6 before radiotherapy to 1.2 after radiation (P=0.0239, Figure 4B). Although radiation therapy upregulates EGFR expression in 4 tumors, the median score of immunohistochemistry staining raised from 2.3 to 2.8, with no statistical significance (P=0.1773, Figure 4C). Probably, this occurred because half of the tumors exhibited EGFR staining with maximum intensity even before radiation therapy. The medium score of ERCC1 staining increased from 1.1 before treatment to 2.0 after radiotherapy (P=0.0294, Figure 4D).

Since mutations may prevent the production of a functional protein, we have investigated possible mutations on exons 5–9 of *TP53* gene through direct sequencing in malignant tissue samples. No mutations were found in tumor samples except for patient #6 (Table 2) who showed two missense mutations (P142S and H179Y). Patient #6 was also one of the few who failed to exhibit ERCC1 and EGFR protein modulation after radiation therapy in tumor tissues. This is notable due to the fact that, since *TP53* gene is not mutated in 9 of 10 cervical tumors, it is not unlikely that p53 protein would display a regulatory role at several genes after radiation therapy, including EGFR and/or ERCC1.

**Table 2.** *TP53* gene mutation status.

Patients	Exon 5	Exon 6	Exon 7	Exon 8	Exon 9
1	WT	WT	WT	WT	WT
2	WT	WT	WT	WT	WT
3	WT	WT	WT	WT	WT
4	WT	WT	WT	WT	WT
5	–	WT	WT	WT	WT
6	Mutant	WT	WT	WT	WT
7	WT	WT	WT	WT	WT
8	WT	WT	WT	WT	WT
9	WT	WT	WT	WT	–
10	WT	WT	WT	–	–

Genomic DNA was extracted from formalin-fixed and paraffin*-*embedded tissues and exons 5–9 of *TP53* gene were amplified by PCR and directly sequenced in both directions. No mutations were found in tumor samples except for patient #6, who presented two missense mutations (P142S and H179Y). WT: wild type. Not analyzed: patient #5: exon 5; patient #9: exon 9; patient #10: exons 8 and 9.

## Discussion

Locally advanced cervical cancer treatment has been based on cisplatin and radiation therapy since 1999 (2), and after almost 20 years there are no major advances on the treatment of this type of cancer. The failure to respond to radiotherapy is a major concern in cervical cancer patients. To this end, studies aiming to detect the molecular mechanisms underlying radioresistance are on high demand. Identification of molecular pathways implicated in the adaptive response of tumor cells to radiation may allow the prediction of treatment outcome and enhance cancer cell killing through employment of selective inhibitors for these pathways.

In the present study, we sought to investigate cell survival and DNA repair proteins potentially implicated in the modulation of radioresistance using both *in vitro* and clinical studies. Indeed, it is well known that several findings from *in vitro* experiments with immortalized cell lines are not confirmed in well-conducted *in vivo* studies. Herein, we observed that although classical cervical cancer cell lines (one radioresistant and one radiosensitive) did not shown any radiation-induced modulation of EGFR, p53, or ERCC1, almost all malignant tissues obtained from cervical cancer patients exhibited a radiation-induced phenotypic change of at least one of these proteins.

The p53 protein expression is not generally observed in tumor cells infected by HPV, as E6 viral oncoprotein causes p53 inactivation by promoting its degradation (11). Curiously, p53 expression was detected at pre-radiotherapy biopsies of cervical cancer. Moreover, there was p53 induction after radiation therapy in 50% of cases. This result suggests that p53 may not be completely inactivated in HPV infected tumor cells. Besides cytotoxic therapies that cause DNA damage, other conditions are capable to induce p53 expression, such as hypoxia (23). It has been demonstrated that hypoxia is a common feature in solid tumors, including cervical cancer (24). Hypoxia is capable to induce p53 expression even in cells infected by HPV through decreasing MDM2 expression (a negative endogenous regulator of p53) and uncoupling the interaction of p53 with the E6 viral protein (25). To date, other groups have already shown p53 expression in cervical tumors by immunohistochemistry (26–28).

These studies, along with ours, suggest that p53 should not be simply considered not expressed in cervical cancer cells. However, the interpretation of p53 induction and its impact in cellular response is not easy to extrapolate. As revised by Ferreira and colleagues (23), the presence of functional p53 could both sensitize or promote resistance in tumor cells exposed to genotoxic therapy, depending on the model utilized and experimental conditions used.

An important regulation between p53 and EGFR was reported previously. Wild type p53 is capable to induce the expression of heparin-binding EGF-like growth factor (HB-EGF), a known EGFR ligand (29). Moreover, a responsive site to wild type p53 was identified at the proximal promoter of the *EGFR* gene (30). These mechanisms reveal a new aspect of p53 function operating beyond apoptosis induction and tumor suppression, what could explain the positive concomitant modulation of p53 and EGFR in 30% of tumors.

EGFR is commonly expressed in normal cells of the basal layer of epithelium but overexpression in tumor cells is closely associated with reduced survival in patients with cervical cancer (6). It is estimated that 90% of cervical carcinomas overexpress EGFR (7), the same magnitude observed here. Furthermore, EGFR has been validated by our group as an important therapeutic target in cervical cancer in preclinical model (18) and in a phase II clinical trial (31). In addition to its physical interaction with components of DNA repair machinery (14,16), EGFR also up-regulates the *ERCC1* gene expression through MAPK signaling pathway (15). This mechanism may explain concurrent induction of EGFR and ERCC1 in 30% of tumor tissues in our study.

ERCC1 is a key protein for DNA repair, especially for nucleotide excision repair (NER). This mechanism is associated with DNA damage repair caused by chemical adducts. In lung cancer, high ERCC1 expression is correlated to platinum chemotherapy resistance (32). However, the ERCC1 role in radioresistance and in repair of DNA damage caused by radiation is recent. Kawashima and colleagues (33) described that ERCC1 silencing promoted sensitization to radiotherapy in bladder cancer. Ahmad and colleagues (12) demonstrated a direct role of ERCC1 in repair of DNA double strand break, the major damage caused by radiation therapy, using *in vitro* and *in vivo* ERCC1 knockout models. Therefore, the regulatory axis p53-EGFR-ERCC1 may be activated in tumor cells exposed to radiation *in vivo*.

Finally, immortalized cell lines have been extensively used to understand the fundamental cancer cell biology mechanisms. Despite its huge contribution to the understanding of cancer biology, many *in vitro* findings fail to reproduce the complex cellular and molecular interactions taking place in individual patients. Our prospective investigational clinical study allowed us to identify potential radioresistance biomarkers that were not modulated in cervical cancer cell lines, highlighting the gap between *in vitro* and *in vivo* experimental models. However, the findings reported in this study need to be confirmed in a larger patient cohort to draw a definitive conclusion on the role of the p53, EGFR, and ERCC1 proteins in radioresistance of cervical carcinoma.
